# Adsorption of the hydrophobic organic pollutant hexachlorobenzene to phyllosilicate minerals

**DOI:** 10.1007/s11356-022-24818-4

**Published:** 2022-12-23

**Authors:** Leonard Böhm, Peter Grančič, Eva Scholtzová, Benjamin Justus Heyde, Rolf-Alexander Düring, Jan Siemens, Martin H. Gerzabek, Daniel Tunega

**Affiliations:** 1 Institute of Soil Science and Soil Conservation, Research Centre for BioSystems, Land Use and Nutrition (iFZ), Justus Liebig University Giessen, Heinrich-Buff-Ring 26, 35392 Giessen, Germany; 2 Institute for Soil Research, Department of Forest and Soil Sciences, University of Natural Resources and Life Sciences Vienna, Peter-Jordan-Straße 82, 1190 Vienna, Austria; 3 Institute of Inorganic Chemistry, Slovak Academy of Sciences, Dúbravská Cesta 9, 845 36 Bratislava 45, Slovakia

**Keywords:** Persistent organic pollutants (POP), Clay minerals, Montmorillonite, Molecular simulations, Halogenated aromatic hydrocarbons, Cations, Hydration enthalpy, Environmental fate

## Abstract

Hexachlorobenzene (HCB), a representative of hydrophobic organic chemicals (HOC), belongs to the group of persistent organic pollutants (POPs) that can have harmful effects on humans and other biota. Sorption processes in soils and sediments largely determine the fate of HCB and the risks arising from the compound in the environment. In this context, especially HOC–organic matter interactions are intensively studied, whereas knowledge of HOC adsorption to mineral phases (e.g., clay minerals) is comparatively limited. In this work, we performed batch adsorption experiments of HCB on a set of twelve phyllosilicate mineral sorbents that comprised several smectites, kaolinite, hectorite, chlorite, vermiculite, and illite. The effect of charge and size of exchangeable cations on HCB adsorption was studied using the source clay montmorillonite STx-1b after treatment with nine types of alkali (M^+^: Li, K, Na, Rb, Cs) and alkaline earth metal cations (M^2+^: Mg, Ca, Sr, Ba). Molecular modeling simulations based on density functional theory (DFT) calculations to reveal the effect of different cations on the adsorption energy in a selected HCB-clay mineral system accompanied this study. Results for HCB adsorption to minerals showed a large variation of solid–liquid adsorption constants *K*_d_ over four orders of magnitude (log *K*_d_ 0.9–3.3). Experiments with cation-modified montmorillonite resulted in increasing HCB adsorption with decreasing hydrated radii of exchangeable cations (log *K*_d_ 1.3–3.8 for M^+^ and 1.3–1.4 for M^2+^). DFT calculations predicted (gas phase) adsorption energies (− 76 to − 24 kJ mol^−1^ for M^+^ and − 96 to − 71 kJ mol^−1^ for M^2+^) showing a good correlation with *K*_d_ values for M^2+^-modified montmorillonite, whereas a discrepancy was observed for M^+^-modified montmorillonite. Supported by further calculations, this indicated that the solvent effect plays a relevant role in the adsorption process. Our results provide insight into the influence of minerals on HOC adsorption using HCB as an example and support the relevance of minerals for the environmental fate of HOCs such as for long-term source/sink phenomena in soils and sediments.

**Supplementary Information:**

The online version contains supplementary material available at 10.1007/s11356-022-24818-4.

## Introduction

Hydrophobic organic chemicals (HOC) are a key group in terms of chemical risk in the environment. In case of halogenated HOCs, hydrophobicity can go along with their persistency, toxicity, and a high bioaccumulation potential. This is especially the case for the group of persistent organic pollutants (POP), a group of substances that have been banned worldwide by the Stockholm Convention in 2001 (United Nations Environment Programme (UNEP) 2020). Owing to their high persistence, their relevance for environmental risk assessment remains high (Wania and Mackay 1996; Ma et al. 2011; Zarfl et al. 2011; Desforges et al. 2018). An example is the former seed treatment agent and fungicide hexachlorobenzene (HCB) that can be found ubiquitously in environmental media (Pohlert et al. 2011; Meijer et al. 2003; Barber et al. 2005). Water solubility of HOCs generally decreases with increasing hydrophobicity, which entails low freely dissolved concentrations in aqueous media as well as their accumulation in solids and biota. Fate and transport of POPs are therefore closely linked with sorption processes at interfaces of solid–aqueous systems.

For HOCs, sorption processes are discussed and investigated with a focus on organic fractions in soils, sediments, and waters, considering the strong hydrophobic interaction of HOCs and organic matter (Karickhoff et al. 1979; Chiou et al. 1986; Pan et al. 2008). While organic matter, quantified as soil organic carbon (SOC), is present in comparatively low percentages in many topsoils depending on ecosystem and land use, and is generally present in even lower contents in subsoils, mineral fractions are quantitatively dominating in most of soils and sediments (e.g., 1.6% SOC in Austrian arable topsoils [median] or rather 0.7–4.9% SOC in German cropland topsoils [benchmark range, between 0.125 and 0.875 quantile]) (Gerzabek et al. 2005; Drexler et al. 2022). Moreover, organic matter appears to accumulate in soils in patchy, cloud-like patterns so that a large fraction of the mineral surfaces is devoid of organic matter coatings (e.g., Ransom et al. 1997; Schweizer et al. 2021).

Mineral composition is highly varying, depending on geological origin and the degree of weathering. Although knowledge of HOC adsorption to mineral phases is still limited compared to organic matter adsorption, interactions between HOCs and minerals were investigated for oxides such as corundum and hematite (Mader et al. 1997) and clay minerals (Hu et al. 2019; Qu et al. 2011; Sadri et al. 2018). For smectites, a family of clay minerals, the effect of exchangeable cations on adsorption was investigated as well for selected organic compounds and cations in laboratory and modeling studies (Qu et al. 2011; Hu et al. 2019; Pašalić et al. 2017). For aromatic compounds, proposed interaction mechanisms include hydrophobic interactions with uncharged mineral surfaces as well as charge-dependent interactions such as free electron–pi electron donor acceptor (n–π EDA) interactions (Pei et al. 2012; Qu et al. 2011). Although several of the studies provide deep insights, they vary widely regarding chemicals and minerals as well as methodical approaches. As a result, systematic approaches with a high variety of minerals or substances are scarce, which can hamper direct comparison of results or their transfer to other systems.

Because environmental contamination by halogenated HOCs such as HCB can be long lasting due to their persistence, knowledge of their environmental fate processes is highly relevant. Given the abundance of mineral surfaces in soils and sediments and based on the limited data on sorption of HOCs to mineral surfaces, we hypothesize that clay minerals have a higher potential to adsorb HOCs than known so far and that this potential is depending on clay mineral type and exchangeable cations. Thus, the aims of this study were (i) to identify the extent of HCB adsorption for a variety of phyllosilicate clay minerals, (ii) to elucidate the influence of exchangeable cations on the extent of HCB adsorption, and (iii) to determine the adsorption mechanism and effect of different exchangeable cations on adsorption by using molecular modeling methods. We determined solid–aqueous adsorption coefficients (*K*_d_) in miniaturized batch equilibrium experiments for HCB and twelve mineral phases as well as a specific montmorillonite (STx-1b) after homoionic saturation with nine different exchangeable alkali and alkaline earth metal cations. By performing DFT calculations, adsorption energies were obtained for the HCB molecule interacting with montmorillonite layer models, in which charges were compensated by different M(I) and M(II) cations. These models corresponded to samples of STx-1b montmorillonite with nine alkali and alkaline earth exchangeable cations.

## Materials and methods

### Chemicals and salts

HCB was purchased as neat substance (purity > 99.5%) from Dr. Ehrenstorfer AG (Augsburg, Germany) and prepared as stock solution in methanol (purity > 99.9%, p.a. quality, Carl Roth GmbH, Karlsruhe, Germany), which was further diluted with methanol yielding working solutions in concentrations between 0.01 and 5 mg L^−1^. Physicochemical properties of HCB are provided in Table S1 in the Supplementary Material (SM).

Alkali and alkaline earth metal salts for homoionic exchange of cations were purchased from Carl Roth in p.a. quality: lithium chloride (LiCl, > 99.0%), sodium chloride (NaCl, > 99.5%), potassium chloride (KCl, > 99.5%), rubidium chloride (RbCl, > 99.0%), and cesium chloride (CsCl, > 99.999%), as well as magnesium chloride (MgCl_2_, > 99.0%), calcium chloride (CaCl_2_, > 99.0%), strontium chloride (SrCl_2_, > 99.0%), and barium chloride (BaCl_2_, > 99.0%). The abbreviation *M*Cl (metal chloride) is used in the following in cases that refer to both alkali and alkaline earth metal chlorides. All water used was of ultrapure quality (Milli-Q Advantage A10 System, Millipore) except for sample treatment for cation exchange that was performed with deionized water (reverse osmosis, RO) from the research facility’s pipeline network.

### Minerals

#### Materials

Clay minerals were purchased from the US Clay Minerals Society (CMS, Chantilly, US-VA) as “source clays” (high-defect kaolinite, KGa-2; low-defect kaolinite, KGa-1b; Ca-montmorillonite, STx-1b; Na-rich montmorillonite, SWy-3; and hectorite, SHCa-1) or rather as “special clays (rock chips)” (montmorillonite “Cheto,” SAz-2; Al-enriched nontronite, NAu-1; Al-poor nontronite, NAu-2; illite, IMt-2; and ripidolite [chlorite], CCa-2). Further mineral phases originated from Ward’s (vermiculite, Transvaal ZA) and Süd-Chemie AG (Bavarian bentonite according to Steudel and Emmerich 2013, thankfully provided by K. Emmerich). Source clays and Bavarian bentonite were used finely ground as delivered “out of the box,” whereas rocks of special clays and vermiculite were broken, afterwards ground by ball mill, and subsequently dry sieved with 200-µm mesh size to remove larger structures that resisted grinding.

#### Cation exchange

The effect of cation type on adsorption was investigated after homoionic cation exchange that was performed with the Ca-montmorillonite “STx-1b” after further sample pretreatment (Tributh and Lagaly 1986a; Poppe et al. 2000; Bergaya and Lagaly 2013). Briefly, treatment with hydrogen carbonate/trisodium citrate dehydrate/sodium dithionite (Merck, Darmstadt, 85%, p. a.) (dissolution of oxides), treatment with acetic acid (Carl Roth, Karlsruhe, Germany, 99.8%, p. a.) (decomposition of carbonates), and treatment with hydrogen peroxide (Merck, Darmstadt, Ph. Eur. Quality) (removal of organic matter) were performed followed by sample washing with deionized water until peptization of particles. Subsequently, the clay fraction (< 2 µm) was separated by five times repeated gravity settling in sedimentation cones and aspiration of the suspended clay fraction after larger particles were sedimented within a timeframe calculated according to Stokes’ law (Lagaly and Dékány 2013; Tributh and Lagaly 1986b). Collected clay suspension was precipitated by addition of CaCl_2_, concentrated by removal of the clear aqueous supernatant, and rinsed with RO-water to remove Cl^−^ ions. Removal was verified by test strips (Quantofix Chloride, Macherey–Nagel). The Ca-precipitated clay was divided and transferred to nine centrifugation tubes for separate cation exchange with the alkali metal cations Li^+^, Na^+^, K^+^, Rb^+^, and Cs^+^ as well as the alkaline earth metal cations Mg^2+^, Ca^2+^, Sr^2+^, and Ba^2+^. For cation exchange, suspensions were centrifuged for 30 min at 710 RCF (Hettich Rotanta 460 R centrifuge). The supernatant was decanted, and instead 25 mL of the specific *M*Cl solutions (0.1 mol L^−1^) were refilled. Samples were then shaken and afterwards centrifuged. This process was performed three times in the same way, apart from the third treatment, where the *M*Cl solution had a concentration of 0.01 mol L^−1^. Cation exchange was verified by elemental analysis. Clay concentrations were calculated from the dry mass content after heating 5 mL of each cation-specific suspension at 105 °C. Clay suspensions were stored in 0.01 mol L^−1^ *M*Cl solution for further usage.

#### Specific surface

Specific surface of bulk minerals and clay fractions after cation modification was detected by physisorption measurements (Quantachrome Quadrasorb evo) according to Brunauer–Emmett–Teller (BET) theory using nitrogen as gaseous adsorbate.

#### Organic carbon

To verify the absence of organic carbon in the minerals, carbon content in minerals was determined (CHNS analyzer Unicube, Elementar Analysensysteme GmbH, Langenselbold, Germany). For samples with total carbon (TC) content above 0.1%, total inorganic carbon (TIC) was derived from carbonate analysis according to Scheibler (DIN EN ISO 10693 2014), and potential total organic carbon (TOC) content was calculated as difference from TC and TIC, which was relevant for SHCa-1 with 0.34% TOC.

### Batch sorption experiments

Sorption experiments with HCB and clay minerals were performed following a modified routine according to test guideline 106 of the Organisation for Economic Cooperation and Development (OECD TG 106) (OECD 2000). For each sorption experiment with pure minerals, constant mineral masses were suspended and spiked with varying HCB concentrations. Briefly, 400 mg (± 2 mg) mineral were weighed to 20-mL brown glass vials, suspended in 10 mL aqueous solution containing 0.01 mol CaCl_2_ L^−1^, and shaken for at least 12 h to ensure sufficient swelling and equilibrium conditions of sorbent and solution. Mineral samples were prepared in duplicates for each HCB concentration. Afterwards, samples were spiked with 4 or rather 5 different HCB concentrations (1, [2,] 3, 4, 5 µg HCB L^−1^) per specific mineral, ensuring environmental relevant concentrations within the water-soluble concentration range of HCB. Samples were then shaken for 24 h to achieve the equilibrium between HCB and sorbents, which was controlled by a preliminary test on adsorption kinetics (Figure S1 in SM). After equilibration, samples were directly centrifuged and transferred to quantitative analysis (compare section “Analysis”). If sorption experiments revealed an especially high adsorption, the specific experiments were repeated with a lower (100 or rather 10 mg) mineral mass. The procedure for clay mineral suspensions with modified cation configuration was basically the same. However, homogenized aliquots of clay suspensions in 0.01 mol L^−1^ *M*Cl were pipetted to brown glass vials instead of dry weighing. Suspensions were homogenized on a horizontal shaker for at least 1 h and vortexed before use. Specific suspension volumes (100–1000 µL) were calculated based on the clay concentration in cation modified clay suspensions after determination of dry mass. Aliquots were transferred by an Eppendorf pipette to the 20-mL headspace vials and diluted to a total volume of 10 mL with 0.01 mol L^−1^ *M*Cl solution of the corresponding cation. *K*_d_ values of experiments with twelve specific minerals are based on one adsorption isotherm each, whereas *K*_d_ values for cation-modified montmorillonite suspensions are given as mean values from two separately performed batch experiments per cation type with the variation given as standard deviation (sd).

### Analysis

Samples were extracted by automated solid-phase microextraction (SPME) following previous studies (Böhm et al. 2016, 2017; Wiltschka et al. 2020) with modifications. Samples and blanks were measured together with an external standard calibration under the same conditions. Briefly, extraction was performed by headspace (HS)-SPME with a 100-µm polydimethylsiloxane (PDMS) fiber (Sigma-Aldrich) at 30 °C for 20 min at a shaking rate of 250 rpm in the agitator (combined heating/shaking device). Prior to extraction, samples were shaken in the agitator for 5 min to allow stabilization of extraction conditions. After extraction, the fiber was directly transferred to the injector of the gas chromatography-mass spectrometry (GC–MS) system, where it was thermally desorbed at 280 °C. Detailed parameters for GC–MS conditions are given in Text and Table S2 (SM). Quality assurance and quality control for SPME extraction were implemented by heating the fiber before extraction and after thermodesorption for 4 min each to prevent carry-over of HCB between samples. Furthermore, blank values of both pure *M*Cl solutions and mineral suspensions without HCB were measured to check for potential carry-over or impurities. The glass vials were used out of the box and were not reused. Fibers were reused and visually controlled for any visible changes and frequently cleaned with methanol.

### Calculation of adsorption

Linear adsorption isotherms were calculated according to OECD TG 106 (OECD 2000), as the ratio between the concentration of adsorbed HCB and the freely dissolved concentration of HCB in the aqueous solution. Particularly, the concentration of adsorbed HCB was calculated according to Eq. 1, presented in OECD TG 106 (OECD 2000).

1 $${C}_{s}= \frac{\left({C}_{0}-{C}_{aq}\right)\times {V}_{0}}{{m}_{min}}$$

*C*_s_ is the concentration of HCB adsorbed to the mineral phase at adsorption equilibrium [μg g^−1^], *C*_0_ is the initial HCB concentration of the aqueous solution in contact with the mineral phase [μg mL^−1^], *C*_aq_ is the mass concentration of HCB in the aqueous phase at adsorption equilibrium [μg mL^−1^], *V*_0_ is the initial volume of the aqueous phase in contact with the mineral [mL], and *m*_min_ is the mass of the mineral phase [g]. *C*_aq_ in all samples was calculated using the calibration curves from external calibration samples. Distribution coefficients (*K*_d_) of HCB adsorption to minerals were derived from the slope of a linear regression between *C*_s_ and *C*_aq_.

### DFT calculations

The calculations were performed by the Vienna Ab initio Simulation Package (VASP) suite (Kresse and Hafner 1993, 1994; Kresse and Furthmüller 1996a, b) applying the Perdew-Burke-Ernzerhof (PBE) functional (Perdew et al. 1996), plane wave basis set (energy cutoff of 400 eV), and using the projector-augmented wave (PAW) atomic pseudopotentials for expressing electronic structure of atoms (Blöchl 1994; Kresse and Joubert 1999). In the calculations, also dispersion corrections of D3 type (Grimme et al. 2010) were included. The *k*-point sampling was limited to *Γ*-point only because of the used large computational cell. In optimization of atomic positions, a force convergence criterion was set to 0.01 eV/Å, and for the SCF convergence, an electronic step condition was 10^−6^ eV.

In DFT calculations, we applied structural models representing one montmorillonite layer (Mnt) with one (for alkali metal compensating ions) or two (for alkaline earth metal ions) octahedral Al^3+^/Mg^2+^ substitutions. These substitutions gave the chemical formulas M(I)_0.125_(Al_3.875_Mg_0.125_)(Si_8_O_20_)(OH)_4_ and M(II)_0.125_(Al_3.75_Mg_0.25_)(Si_8_O_20_)(OH)_4_, respectively. The calculations were performed using periodic slab models with lateral *a* and *b* lattice vectors of 20.97 and 18.18 Å, respectively. In the third dimension, a vacuum of about 20 Å was imposed to eliminate interactions between periodic images in the *c* direction. These vectors were obtained from the optimization of the computational cell of the Na-Mnt single layer and then kept fixed for all models (including HCB molecule) with M(I) and/or (MII) cations, respectively.

The compensating cation was placed above a center of the ditrigonal hole in the tetrahedral sheet nearby the octahedral substitution. The HCB molecule was placed above the compensating cation in a parallel configuration with the montmorillonite surface. No environmental effects (e.g., solvent) were considered in the calculations. All atomic positions were relaxed using fixed unit cell parameters. To calculate adsorption energies, the isolated HCB molecule and the montmorillonite layer were optimized as well using the same conditions for the calculations.

Further, we also performed additional calculations on HCB-M(I)/M(II) complexes using the molecular program Turbomole (v. 7.2) (Ahlrichs et al. 1989; Arnim and Ahlrichs 1998) to reveal pure interactions of cations with HCB. The calculations were performed with the same PBE functional combined with the RI approximation (Resolution of Identity) (Eichkorn et al. 1997) and D3 dispersion correction scheme (Grimme et al. 2010) and the atomic basis set of def2-TZVP quality (Triple-Zeta Valence Polarization) (Weigend et al. 1998, 2003). Solvent effect in the Turbomole calculations was approximated implicitly by using the COSMO model (COnductor-like Screening MOdel) (Klamt and Schüürmann 1993).

## Results and discussion

### HCB adsorption to clay minerals

Adsorption experiments with HCB and reference clay minerals showed a large variation of adsorption covering several orders of magnitude (log *K*_d_ 0.9–3.3, Table 1). The results illustrate the highly varying extent of HCB adsorption to pure mineral phases. Generally, adsorption to pure mineral phases was lower in our experiments comparing to the well-known strong sorption of HCB to organic matter (Böhm et al. 2016; Jepsen et al. 1995; Mackay et al. 2006, Müller‐Wegener 1981) (exemplary values provided in SM, Table S1). However, our results show strong adsorption to selected minerals (log *K*_d_ values of 2.6–3.3, Table 1) and demonstrate that the adsorption to selected phyllosilicates is comparable with the sorption to solid phases with a relevant content of natural organic matter. For example, our own measurements showed that the HCB sorption to river sediments with 1.7–6.7% TOC yielded log *K*_d_ values in a range of 2.8–3.5 (own unpublished data). Our mineral selection included phyllosilicate minerals that vary widely in their characteristics such as structure, composition, specific surface area (SSA), or layer charge. Adsorption strength was neither correlated with SSA (*R*^2^ < 0.10) nor cation exchange capacity (*R*^2^ < 0.15) nor with TOC (detected only in one mineral phase). However, sorption strength can be roughly differentiated according to phyllosilicate types. Low adsorption was observed for kaolinite as a representative of the 1:1 type (log *K*_d_ ≈ 1.0). HCB adsorption to the 2:1 phyllosilicates with exchangeable interlayer cations (smectites, vermiculite, and illite) varied in a wide range (log *K*_d_ ≈ 0.9–2.8, Table 1). The highest adsorption was detected for chlorite (log *K*_d_ = 3.3), referred to as a 2:1:1 type. Chlorite has a hydroxide layer sandwiched between TOT (2:1) layers. Substitution of M^2+^ by M^3+^ cations in the hydroxide layer can lead to a positive charge that compensates a negative charge of 2:1 layers (Blume et al. 2016). This reduction of charge in the structure of chlorite CCa-2 might have led to the existence of local neutral sites at the surface and, thereby, to a higher affinity of HCB towards the adsorbent. However, based on the structural differences within investigated minerals, the number of adsorbents in the set is neither sufficient to derive significant correlations between adsorption strength and specific mineral parameters nor to derive mechanistic relations. For example, determination of specific surface area by N_2_ adsorption (BET) resulted in values between 12 and 96 m^2^ g^−1^ for the minerals listed in Table 1 but did not explain varying HCB adsorption (Table S3 in SM). However, results for the mineral selection given in Table 1 allow pointing to the huge variety of mineral characteristics and the necessity to further investigate HOC–clay mineral interactions in a detailed and systematic way.

**Table 1:** Adsorption of HCB (1–5 µg L^−1^) in the presence of native mineral materials (1–40 g L^−1^). *R*^2^ is given as measure of isotherm linearity

Mineral	Origin^a^	*K*_d_	log *K*_d_	*R*^2^	*RMSE (n)*^*b*^
Kaolinite (high-defect)^c^	CMS, KGa-2	12	1.10	0.9883	3.1 (4)
Kaolinite (low-defect)^c^	CMS, KGa-1b	11	1.06	0.9827	0.9 (4)
Smectite (Ca-montmorillonite)^c^	CMS, STx-1b	7	0.85	0.9906	1.9 (4)
Smectite (montmorillonite “Cheto”)^c^	CMS, SAz-2	75	1.87	0.9997	1.1 (5)
Smectite (Na-rich montmorillonite)^c^	CMS, SWy-3	389	2.59	0.9983	3.4 (4)
Smectite (“calcigel”)^c^	Bavaria, Germany	664	2.82	0.9988	11.5 (5)
Smectite (Al-enriched nontronite)^c^	CMS, NAu-1	12	1.08	0.9991	0.7 (4)
Smectite (Al-poor nontronite)^c^	CMS, NAu-2	182	2.26	0.9997	1.2 (5)
Hectorite^c^	CMS, SHCa-1	566	2.75	0.9950	5.6 (5)
Illite^d^	CMS, IMt-2	142	2.15	0.9951	5.3 (4)
Vermiculite^d^	Transvaal (SA)	565	2.75	0.9984	12.1 (4)
Chlorite (ripidolite)^e, f^	CMS, CCa-2	2 076	3.32	0.9715	410.6 (4)

^a^CMS: Source Clays Repository of the US Clay Mineral Society.

^b^Root-mean-square error of C_s_/C_aq_ regression (n: number of test substance concentrations per isotherm, each prepared in duplicates).

^c^Experiment with 40 g sorbent L^−^^1﻿^

^d^Experiment with 10 g sorbent L^−^^1﻿^

^e^Experiment with 1 g sorbent L^−^^1﻿^

^f^For reason of comparability, the data for HCB adsorption to chlorite are given as well based on the linear relation, although the adsorption isotherm rather matches a Freundlich relation (*K*_F_ = 2 396, Freundlich exponent (1/n) = 0.57, *R*^2^ = 0.9934).

### Cation-modified montmorillonites

**Fig. 1:**
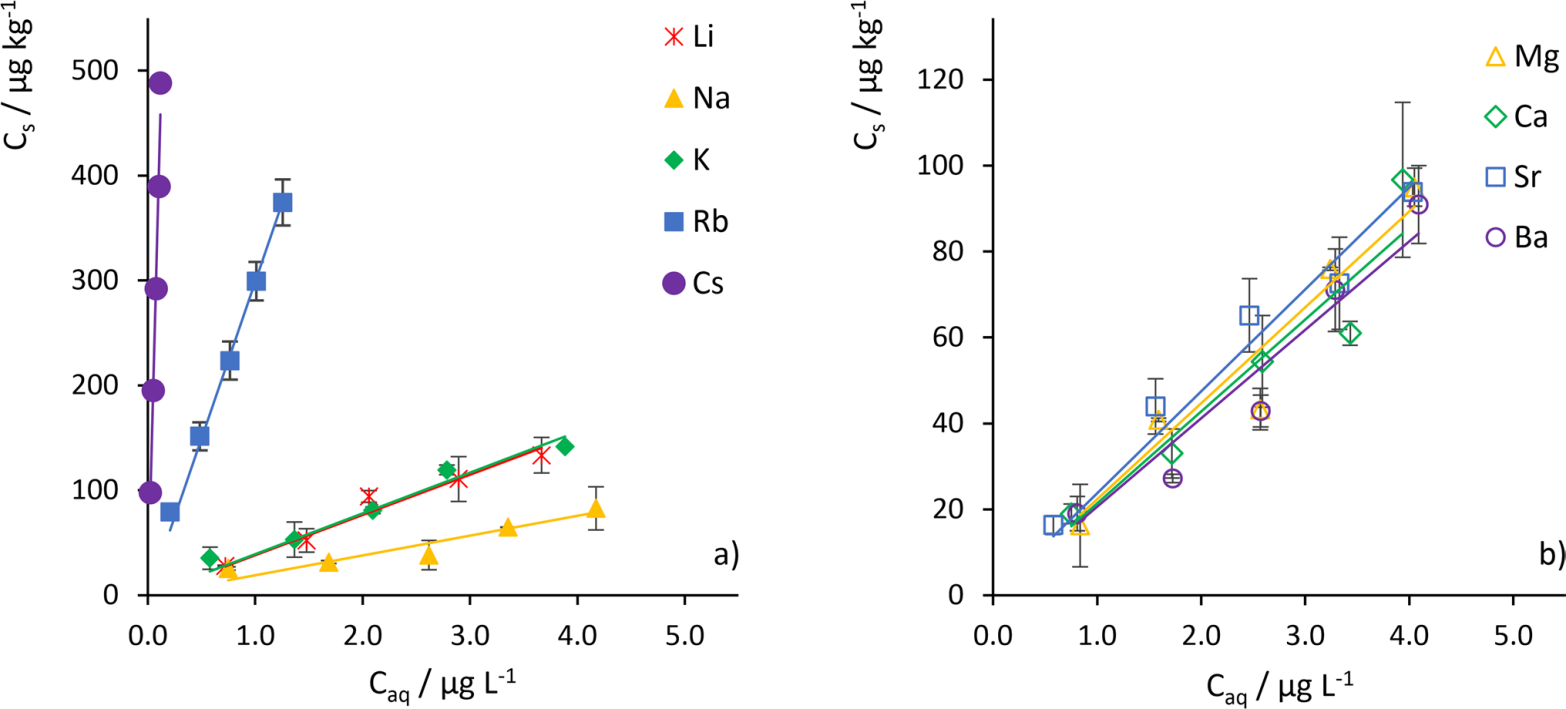
Adsorption isotherms for the interaction of HCB with the cation-modified clay mineral montmorillonite STx-1b. Homoionic cation exchange was performed with **a** alkali metal cations, and **b** alkaline earth metal cations (clay mineral concentration: 10 g L^−1^; particle size fraction: < 2 µm, HCB concentration: 1–5 µg L^−1^). Note different scales of y-axes. All isotherms in Fig. 1b are in the range of adsorption to Na^+^-modified montmorillonite in Fig. 1a

**Fig. 2:**
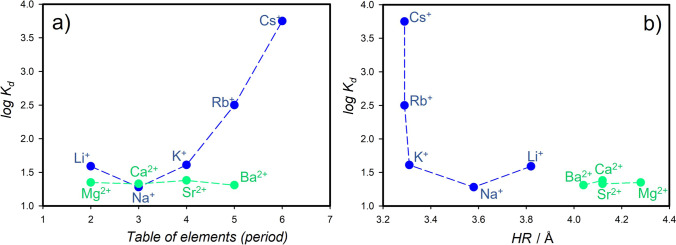
Adsorption of HCB in the presence of cation-exchanged montmorillonite (CMS, STx-1b). Exchange was performed with alkali (blue circles) and alkaline earth (green circles) metals. Relation between **a** log *K*_d_ and the period of table of elements and **b** log *K*_d_ and hydrated radius (*HR*). Values of *HR* are adopted from Nightingale (1959)

Further adsorption experiments were conducted with a montmorillonite (CMS, STx-1b) that was fractionated (< 2 µm) and modified in its cation configuration. Samples, labeled as *M*-Mnt, were prepared with alkali elements, M(I): Li^+^, Na^+^, K^+^, Rb^+^, and Cs^+^, and alkaline earth elements M(II): Mg^2+^, Ca^2+^, Sr^2+^, and Ba^2+^. The results revealed a significant difference between the effects of M(I) and M(II) exchangeable cations on the HCB adsorption (Figs. 1 and 2, log *K*_d_ values in Table 2). Whereas M(I)-Mnt samples had broadly varying adsorption isotherms resulting in log *K*_d_ values in a wide range of 1.28–3.75 (Fig. 1a, Table 2), adsorption isotherms of the M(II)-Mnt samples showed practically no sensitivity on the type of the M(II) cation (log *K*_d_ values vary in a narrow range 1.31–1.38, Fig. 1b, Table 2). Moreover, the variation of log *K*_d_ isotherm replicates exceeded differences between log *K*_d_ values for the M(II)-Mnt samples. For the M(I)-Mnt samples with cations from the group of alkali metals, we observed increasing adsorption with increasing atomic number (from Na^+^ to Cs^+^, Fig. 2a). The exception is the Li^+^ cation, for which the adsorption was stronger than the adsorption for the Na-Mnt sample and comparable to the adsorption of the sample with K^+^ (log *K*_d_ (Li-Mnt) of 1.59 vs. 1.61 of K-Mnt, Table 2). The adsorption to the Na-Mnt sample was similar as for all M(II)-Mnt samples (log *K*_d_ of 1.28 vs. 1.31–1.38, Table 2). The strongest adsorption was observed for the Rb-Mnt and Cs-Mnt samples with log *K*_d_ values of 2.50 and 3.75, respectively.

**Table 2:** Experimental adsorption coefficients (log *K*_*d*_) for the adsorption of HCB to the fractionated (< 2 µm) and M(I)/M(II)-modified montmorillonite sample STx-1b, DFT (PBE-D3) calculated adsorption energies (*E*_ads_), *d*1 (HCB to cation), and *d*2 (cation to surface) distances in the HCB-montmorillonite models as well as effective ionic radii (*IR*) for M(I)/M(II) ions in aqueous solution, ionic charge densities (*ρ*, calculated from formal ionic charge and *IR*), hydrated radius (*HR*), and standard hydration enthalpies (*H*_*hyd*_)

M^+/2+^	This work				*IR*^b^/Å	*HR*^*c*^/Å	*ρ*/ |*e*| Å^−3^	*H*_hyd_^d^/kJ mol^−1^
	log *K*_d_ ± sd	*E*_ads_/kJ mol^−1^	*d*1/Å	*d*2^a^/Å				
Li	1.59 ± 0.08	− 74.0	3.411	− 0.077 (− 0.123)	0.71	3.82	0.667	− 531
Na	1.28 ± 0.09	− 75.9	2.956	0.482 (0.536)	0.97	3.58	0.262	− 416
K	1.61 ± 0.04	− 31.1	3.727	1.383 (1.309)	1.41	3.31	0.085	− 334
Rb	2.50 ± 0.11	− 29.7	3.645	1.599 (1.583)	1.50	3.29	0.071	− 308
Cs	3.75 ± 0.27	− 23.8	3.417	1.856 (1.906)	1.73	3.29	0.046	− 283
Mg	1.35 ± 0.01	− 83.7	3.230	− 0.154 (− 0.234)	0.70	4.28	1.392	− 1949
Ca	1.33 ± 0.08	− 96.2	3.026	0.311 (0.159)	1.03	4.12	0.437	− 1602
Sr	1.38 ± 0.05	− 76.5	3.222	0.702 (0.536)	1.25	4.12	0.244	− 1470
Ba	1.31 ± 0.06	− 70.5	3.027	1.026 (1.018)	1.35^e^	4.04	0.194	− 1332

^a^Number in () is *d*2 distance for the bare surface.

^b^Ionic radius (Marcus 1988).

^c^Hydrated radius (Nightingale 1959).

^d^Hydration enthalpy (Marcus 1987).

^e^From Shannon (1976)

**Fig. 3:**
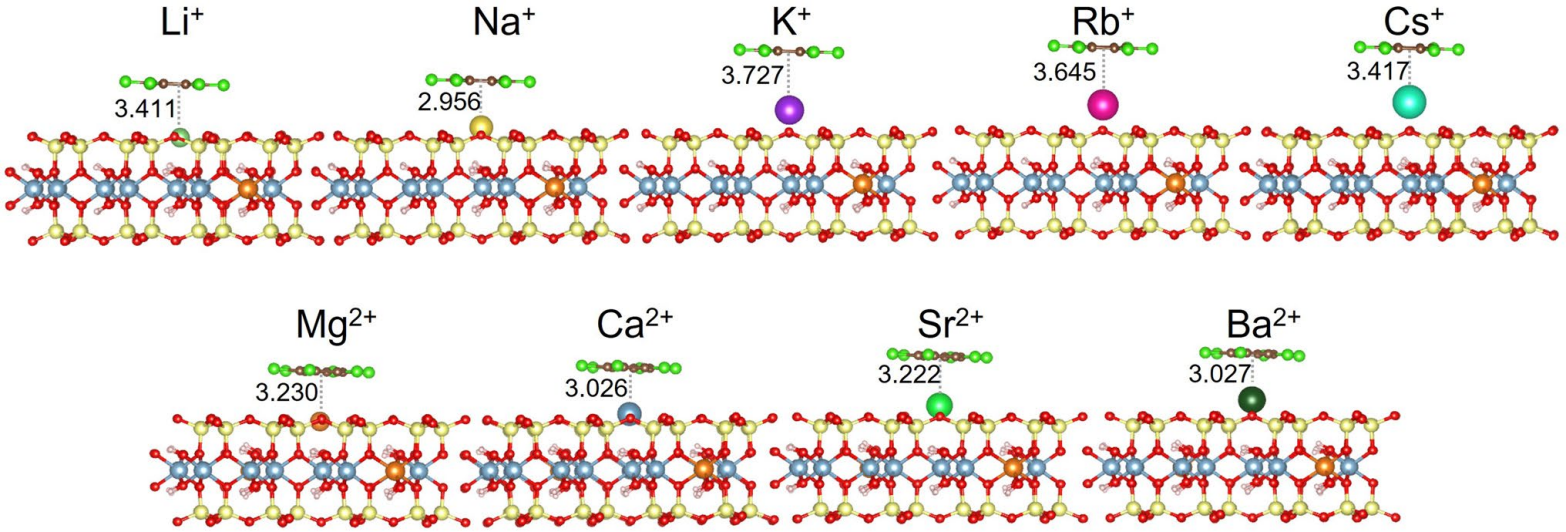
PBE-D3-optimized geometries of HCB molecule adsorbed on single-layer models of montmorillonite with 5 alkali and 4 alkaline earth element groups. Numbers represent *d*1 distance in Å (Table 2)

To explain observed trends of the adsorption for the M-Mnt samples, several factors must be taken into account. If we suppose that the adsorption mechanism is mainly represented by cation-π interactions of cations compensating the excess layer charge at outer surfaces of montmorillonite particles, cationic size represented by ionic radius (*IR*) and hydration of cations can play a dominant role. From the potential factors affecting adsorption, we can exclude the SSA as a causal parameter because cation-modified samples had similar SSA values (54–80 m^2^ g^−1^, Table S4 in SM). In the supposed cation-π adsorption mechanism, outer particle surfaces of montmorillonite are represented dominantly by the basal (001) surface, where the surface cations are sandwiched between this surface and the aromatic ring of the HCB molecule in a parallel configuration with the surface. Structural models representing this adsorption mechanism (Fig. 3) were used in the DFT calculations.

**Fig. 4:**
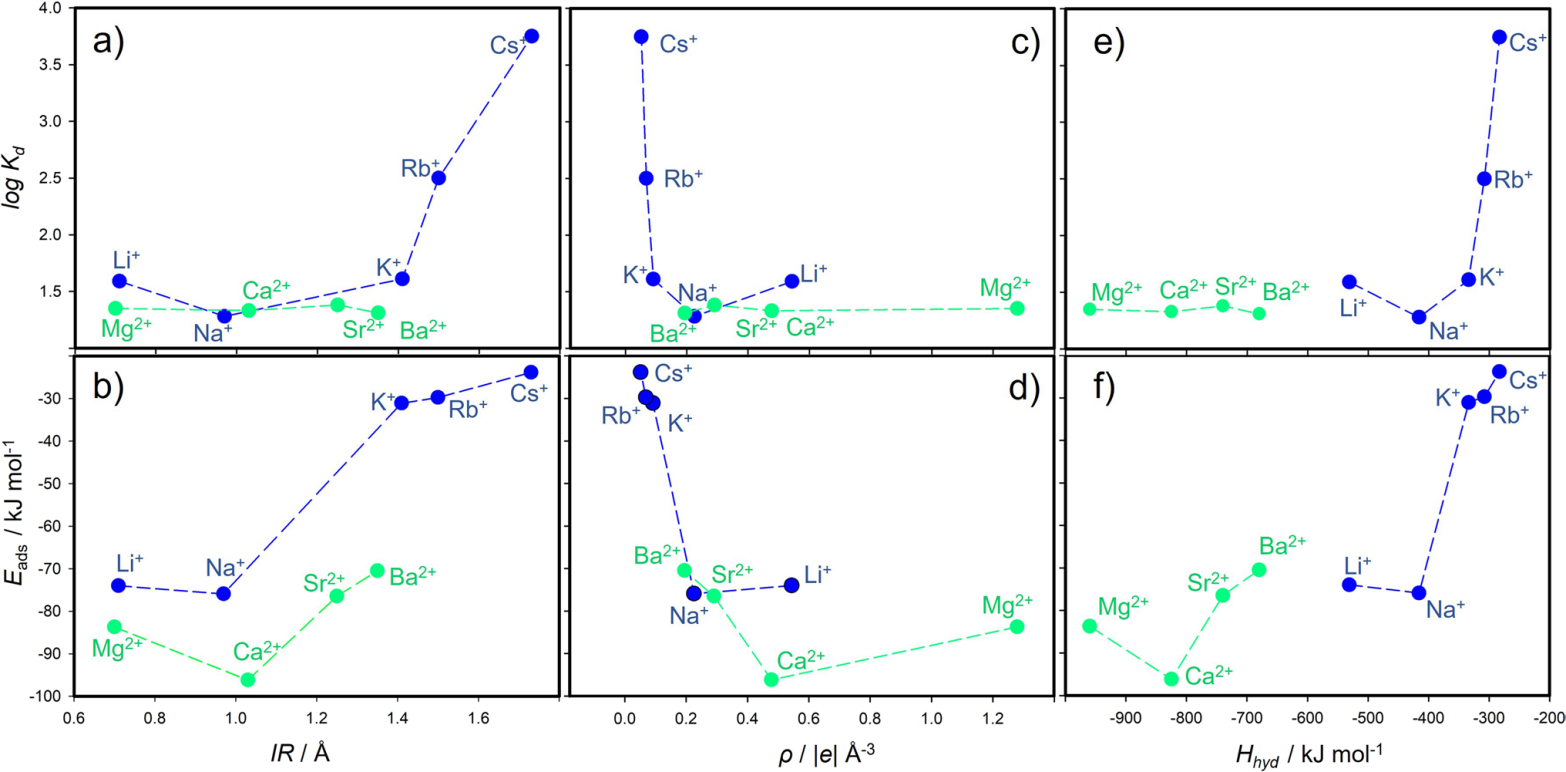
Relations of experimentally determined log *K*_d_ values and DFT-calculated adsorption energy (*E*_ads_) of HCB with respect to ionic radius (IR), ionic charge density (ρ), and hydration enthalpy (*H*_hyd_) of M(I)/M(II) cations (for better scale, *H*_hyd_ for M(II) are normalized to charge)

However, ionic radius itself is not enough to explain the observed different trends in adsorption for M(I) and M(II) cations (Fig. 4a) because their radii are comparable (*IR* in Table 2). Note that there are several definitions of *IR*, and for our purposes, we took the definition by Marcus (1988) for cations in ionic solutions. As alkali metal cations are singly and alkaline earth metal cations are doubly positively charged, charge density and cation distribution at the surface could affect the adsorption. In the montmorillonite–solution systems, cations presented at outer surfaces undergo at least partial hydration affecting the surface hydrophobicity. Therefore, hydrated radius (*HR*) of cations, which depends on the charge density, could be an appropriate parameter to explain varying adsorption in alkali and alkaline earth metal groups. Figure 2b shows a relation between experimental log *K*_d_ and *HR*; however, a quantitative correlation is not evident. While Rb^+^ and Cs^+^ as well as Ca^2+^ and Sr^2+^ do not differ within their hydrated radius, they do in their charge density (Table 2).

DFT calculations performed on the adsorbed single HCB molecule on the nine models represented by a single montmorillonite layer with different M(I) and M(II) compensating cations (Fig. 3) helped to analyze the effect of ionic type on the strength of adsorption of HCB even though these calculations did not include a solvent (hydration) effect on the adsorption. The results from the DFT geometry optimization of HCB on M(I)/M(II)-Mnt models are summarized in Table 2. This table contains computed adsorption energies, *E*_ads_, and perpendicular distances of the center of mass of the HCB molecule to the compensating cation (*d*1, shown also in Fig. 3) and of this cation to the plane of the basal surface oxygen atoms of the montmorillonite layer (*d*2).

**Fig. 5:**
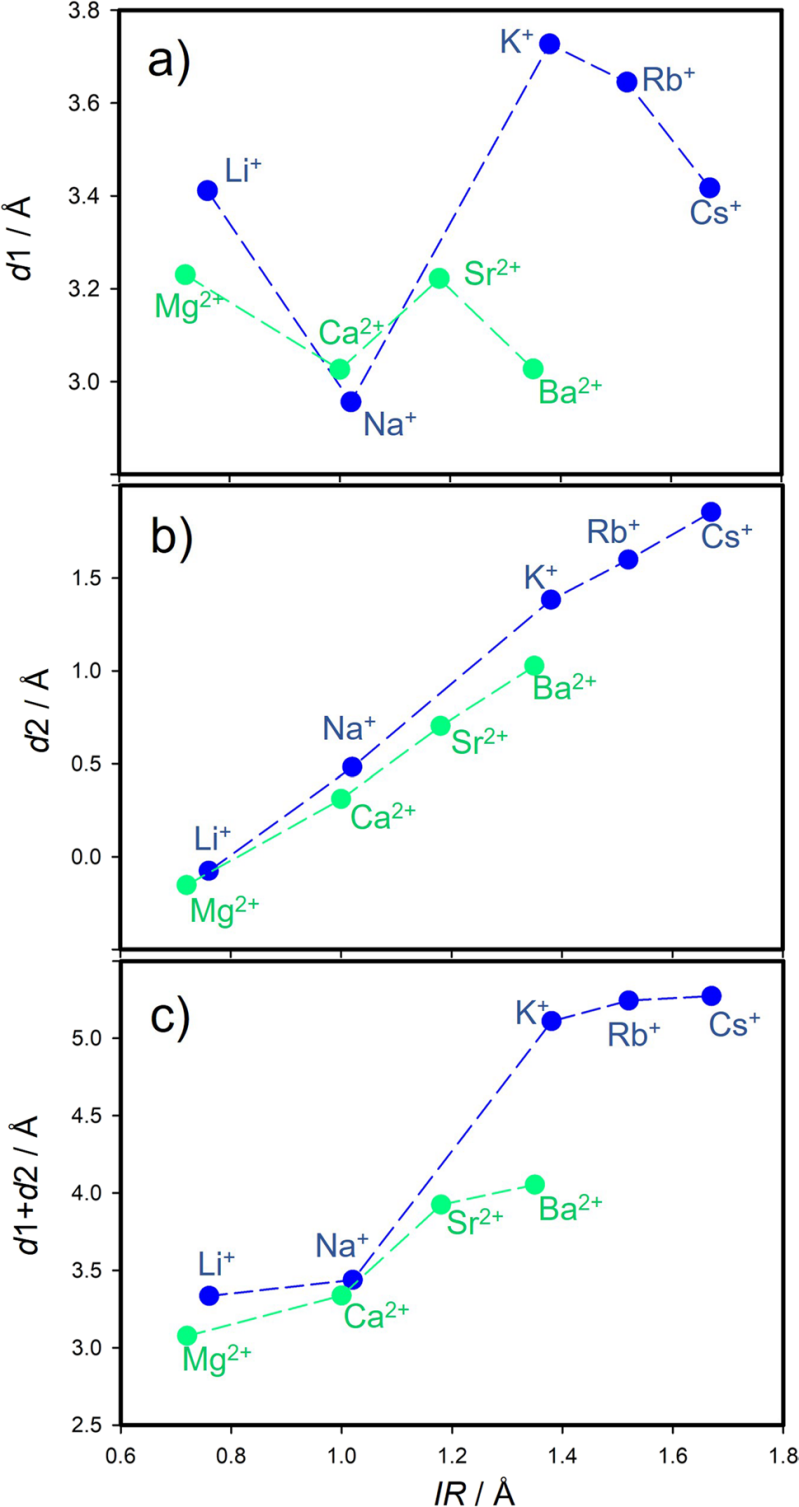
DFT-optimized distances between M(I)/M(II) cation and the center of mass of HCB molecule (*d*1), cation and the place of basal surface oxygen atoms of the montmorillonite layer (*d*2), and their sum *d*1 + *d*2

**Fig. 6:**
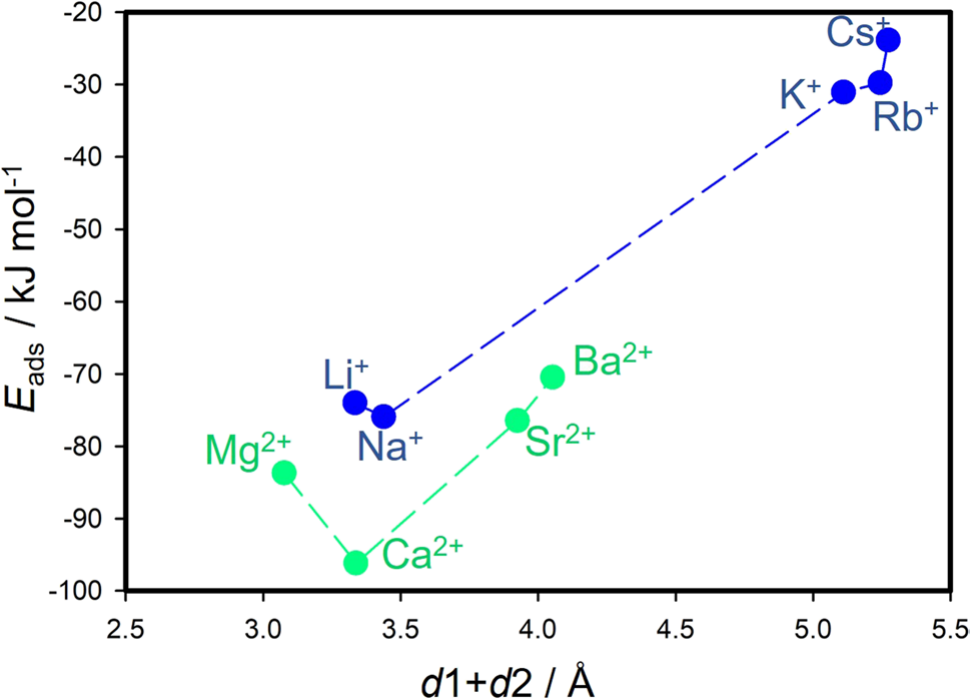
Relation between calculated adsorption energy of the HCB molecule and its distance to the plane of the basal surface oxygen atoms of the montmorillonite layer

**Fig. 7:**
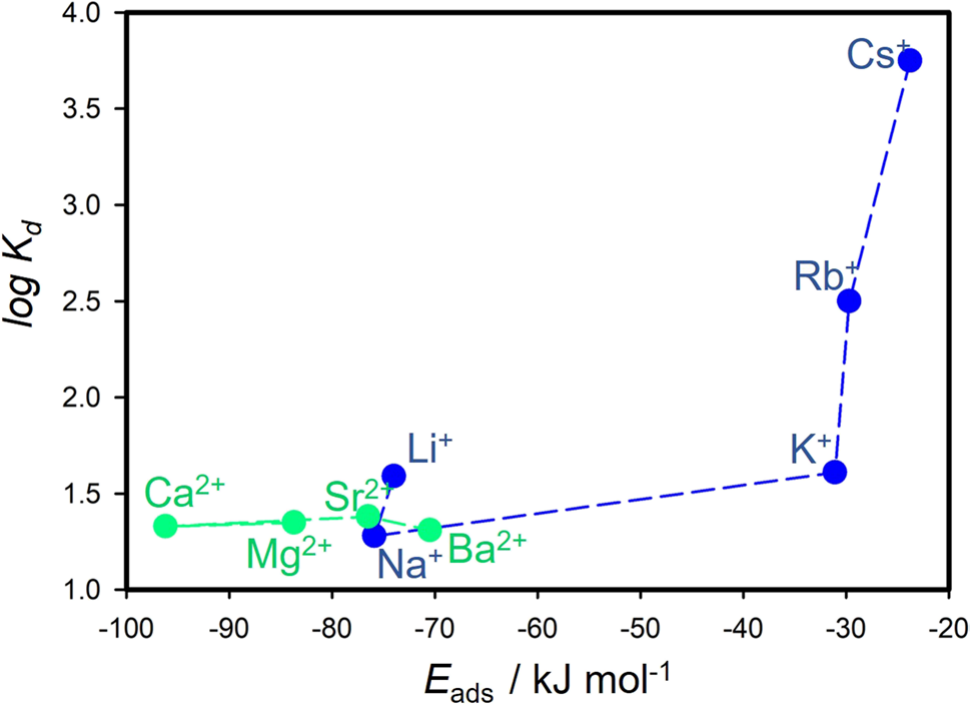
Relation between measured log *K*_d_ values and calculated adsorption energies, *E*_ads_

From the optimized geometries (Fig. 3), it was observed that the HCB molecule has always a configuration with the molecular plane parallel to the surface of the montmorillonite layer. The compensating cation is always located in the center of the ditrigonal hole of the silicate sheet. The HCB molecule is positioned in a way that the compensating cation is coordinated by the aromatic ring. Figure 5 shows a relation of *d*1 and *d*2 distances to the *IR*. Evidently, the *IR* determines how far the ion is displaced (*d*2) from the plane of the basal oxygen atoms (O_b_). Table 2 also provides an overview of the distances of the cations to the O_b_ plane for the bare montmorillonite surface (values in ()). The ions with the smallest *IR* (Li^+^ and Mg^2+^) are embedded in the ditrigonal hole (negative *d*2). Trends of the *d*2 distance are nearly linear with respect to *IR* (Fig. 5b), differing from the trends of the *d*1 values (Fig. 5a). The *d*1 trend shows the zig-zag behavior with a paradox that *d*1 for the smallest M(I) cation Li^+^ is almost the same as the *d*1 distance for the largest Cs^+^ cation. Consequently, *IR* strongly affects the overall distance (*d*1 + *d*2) of the HCB molecule from the O_b_ plane (Fig. 5c). The shortest *d*1 + *d*2 distances were observed for Li^+^ and Mg^2+^, respectively. However, the shortest overall distance does not mean the strongest adsorption of the HCB molecule (Fig. 6). The largest calculated adsorption energies (*E*_ads_, Table 2) were found for Na^+^ and Ca^2+^ cations and not for Li^+^ and Mg^2+^ as one could expect. These two smallest cations are deeply embedded in the ditrigonal hole of the montmorillonite layer model (see negative *d*1 distances in Table 2); thus, the HCB molecule is not in an optimal distance from the basal surface due to a repulsion between large Cl atoms of the HCB molecule and O_b_ atoms. This explains why *E*_ads_ are smaller (in absolute value) for Li^+^ and Mg^2+^ in a comparison to Na^+^ and Ca^2+^, respectively. This could also explain an observed anomaly for the measured log *K*_d_ values for the Li-Mnt sample (Fig. 4a and b). On the other hand, the smallest adsorption energies (in absolute value) were found for the monovalent ions with the large *IR* (K^+^, Rb^+^, and Cs^+^) and, correspondingly, with the large *d*1 + *d*2 distances being clearly separated in Fig. 6 from the rest of cations. A similar separation is also observed in the relation between calculated *E*_ads_ and *IR* (Fig. 4b). Comparing trends for log *K*_d_ and *E*_ads_ with respect to *IR*, there is a certain similarity (Fig. 4a and b). For small- to medium-sized cations (except *E*_ads_ for K^+^), both parameters are less sensitive to the *IR*, more evidently for log *K*_d_ of all M(II) cations. The largest monovalent cations Rb^+^ and Cs^+^ differ from the rest of cations. Supposing that these cations have small adsorption energies, from the model perspective, log *K*_d_ values for Rb- and Cs-Mnt should be also relatively small. However, the opposite trend was observed, and samples with these two cations had the highest log *K*_d_ values. Therefore, there is no straightforward relationship, in which *K*_*d*_ increases with increasing adsorption energy *E*_ads_ (Fig. 7).

Thus, *IR* is an important factor affecting the strength of adsorption of HCB for M(I) cations but not for M(II) cations. Similar trends as shown in Fig. 4a and b were observed also in case if the cation charge density was taken into account (Table 2, Fig. 4c and d). Even though the Mg^2+^ cation has the largest charge density of all cations, there is no evident impact on log *K*_d_ or calculated *E*_ads_.

To show how *IR* is related to the interaction between cations and the aromatic ring of the HCB molecule, we performed additional molecular calculations on the pure complexes of the HCB molecule with all cations. This allowed to separate the effect of cation interactions with the montmorillonite surface. These results are presented in Table 3.

**Table 3:** Calculated interaction energies (in kJ mol^−1^) of HCB molecule with all cations in the gas phase (*E*_int_) and in aqueous solvent (*E*_int_^hyd^, COSMO calculations—see computational details). *d*1 and *d*1^hyd^ are distances (in Å) between cation and the center of the HCB ring in the HCB-M complex in the gas phase and in solvent. *G*_calc_^hyd^ (in kJ mol^−1^) is COSMO calculated solvation free energy of M(I) and M(II) cations, *G*_exp_^hyd^ (in kJ mol^−1^) is experimental solvation free energy (*T* = 298 °C) (Burgess 1978). In COSMO calculations, effective Born radii (*IR*_Born_) for cations were used in order to reproduce free energies of solvation, taken from Babu and Lim (1999)

M^+^/M^2+^	*E*_int_	*d*1	*E*_int_^hyd^	*d*1^hyd^	*G*_calc_^hyd^(M)	*G*_exp_^hyd^(M)	*G*_calc_^hyd^(HCB-M)	*IR*_Born_
Li	− 100.0	1.890	64.6	2.273	− 489.3	− 510.7	− 372.7	1.39
Na	− 61.0	2.462	15.3	3.319	− 402.4	− 410.8	− 358.9	1.69
K	− 44.1	2.959	0.2	3.915	− 340.1	− 337.1	− 318.1	2.00
Rb	− 40.7	3.215	− 2.1	4.143	− 316.4	− 315.8	− 297.9	2.15
Cs	− 38.7	3.357	6.0	3.778	− 290.7	− 283.6	− 267.2	2.34
Mg	− 461.5	1.921	244.4	2.187	− 1915.8	− 1905.4	− 1382.0	1.42
Ca	− 323.8	2.291	165.5	2.628	− 1572.5	− 1592.9	− 1231.2	1.73
Sr	− 250.6	2.504	128.4	2.917	− 1416.9	− 1446.9	− 1164.8	1.92
Ba	− 215.5	2.694	203.5	3.016	− 1307.9	− 1318.1	− 976.2	2.08

Calculated interaction energies in gas phase, *E*_int_, document the strong cation-π interactions, specifically for divalent M(II) cations, which energies are more than 4 times larger than for monovalent M(I) cations. Such big difference is not observed for the calculated adsorption energies (*E*_ads_, Table 2) of HCB on M-Mnt layer models, where *E*_ads_ are comparable for alkali and alkaline earth elements. As expected, gas–phase interaction energies showed a monotonic decreasing trend with increasing atomic number of both sets of cations (thus, with increasing *IR*). Distances *d*1 show the opposite trend—their increase with the increasing atomic number.

The calculations on molecular HCB-cation models allowed to include solvent effect using the implicit COSMO model (Klamt and Schüürmann 1993) to calculate hydration free energies, *G*_calc_^hyd^ (Table 3). In the COSMO calculations, we used effective Born radii for cations to reproduce the hydration free energy (*G*_calc_^hyd^(M) and *G*_exp_^hyd^(M) in Table 3). The agreement between calculated and experimental hydration free energies is good. The calculated hydration free energies of the HCB-M complexes, also collected in Table 3, are smaller than the corresponding hydration free energies of isolated cations due to effective screening of the part of the spherical cation by the hydrophobic HCB molecule (its calculated hydration free energy is small, *G*_calc_^hyd^(HCB) =  − 8.1 kJ mol^−1^). The difference between *G*_calc_^hyd^(HCB-M) and *G*_calc_^hyd^(M) predominantly decreases with the increasing atomic number for both M(I) and M(II) sets, respectively. The most important observation from the COSMO calculations is that the hydration effect strongly destabilizes the HCB-M complexes being evidenced by the calculated interaction energies *E*_int_^hyd^ (Table 3). Except the Rb^+^ cation, all calculated interaction energies are positive. Moreover, for M(II) cations, the destabilization of the HCB-M complex is very strong.

The results for the HCB-M complexes confirmed that the hydration is also an important factor for the stability of adsorbed M-Mnt complexes. In addition, for these surface complexes, hydration will represent a strong destabilization factor for the HCB adsorption to montmorillonite. Table 2 summarizes, amongst others, data on standard hydration enthalpy (*H*_hyd_) for M(I) and M(II) and cations in aqueous solution collected from the literature (Marcus 1987, 1988; Nightingale 1959). The relationship between log *K*_d_/*E*_ads_ and *H*_hyd_ (Fig. 4e and f) shows a similar trend as the relationship to *IR* (Fig. 4a and b). However, *H*_hyd_ for M(II) cations separate from *H*_hyd_ for M(I) cations, even though *H*_hyd_ for M(II) are normalized to charge. The relationships between log *K*_d_/*E*_ads_ to individual parameters such as *IR*, ρ, or *H*_hyd_ (Fig. 4) are similar as on x-axis is always a parameter exhibiting a monotonic, nearly linear trend (decreasing or increasing). From the computational perspective on gas-phase adsorption, one could expect that larger cation hydration means larger surface hydrophilicity, thus larger destabilization effect on the HCB adsorption, whereas the smaller *IR* (in other words, larger charge density) can enhance the adsorption (see *E*_int_ in Table 3). Therefore, there is an interplay between *IR* and hydration enthalpy of cations and how they are balanced.

**Fig. 8:**
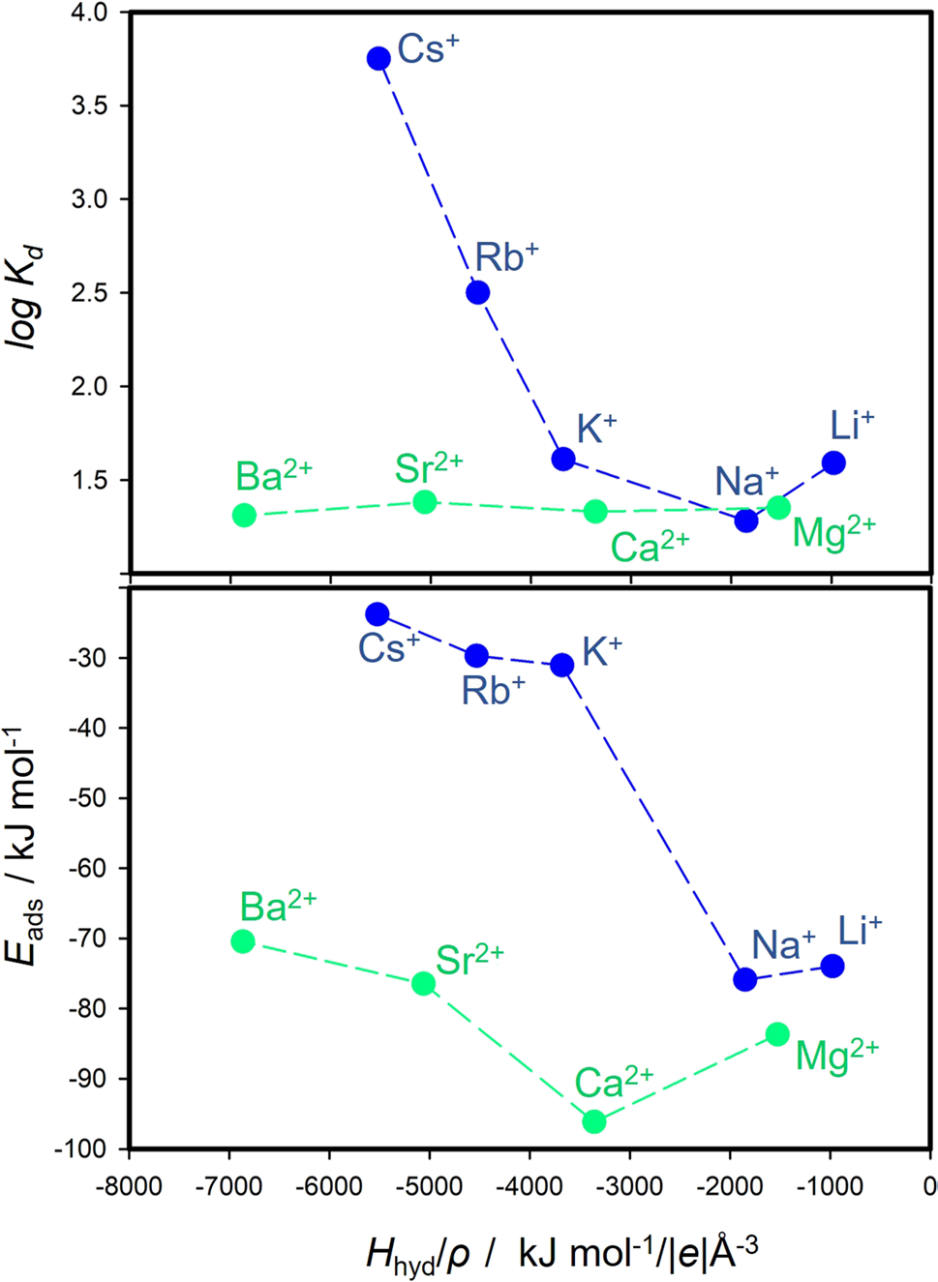
Relationships of experimental log *K*_d_ and calculated *E*_ads_ to complex parameter *H*_hyd_/ρ

To include both effects, *IR* and *H*_hyd_, in one parameter, we related *E*_ads_ and log *K*_d_ with respect to *H*_hyd_ divided by the ionic charge density, ρ (Fig. 8). Evidently, relationships *E*_ads_–*H*_hyd_/ρ and log *K*_d_ –*H*_hyd_/ρ are comparable for M(II) metals. The trends for M(I) metals are seemingly similar. However, a higher adsorption energy for species with higher log *K*_d_ (Rb^+^ and Cs^+^) would be expected—what is not the case as shown in Fig. 8. A reason for this contradiction might be the role of water molecules in the hydration of species in adsorption experiments. This effect was only partially reflected in calculations on models of HCB-M complexes where polarizable continuum models were used. The description of the hydration effect in detail (e.g., formation of the first hydration shell) requires the inclusion of the solvent effect explicitly by adding water molecules to the modeled systems. However, such large and complex models require a different computational strategy such as molecular dynamics that is much more computationally demanding at the DFT level. This approach will be considered in our upcoming work.

## Conclusions

Results from adsorption experiments with native clay minerals clearly showed a broad range of HCB adsorption strength to various clay minerals (log *K*_d_ 0.9–3.3). The results indicated that minerals could play a relevant role as a pollutant sink in environmental fate processes even for hydrophobic chemicals. Experiments with cation-exchanged montmorillonite samples revealed the influence of the cation configuration of clay mineral samples on HCB adsorption strength. HCB adsorption was similar for most alkali and alkaline earth metal cations (log *K*_d_ 1.3–1.6), except for modifications by Rb and Cs that led to highly increased HCB adsorption (log *K*_d_ 2.5 and 3.8, respectively). Due to the rare abundance of Rb and Cs in environmental media, this is of minor relevance from an environmental fate perspective but is of high relevance for the general understanding of interaction mechanisms. The results obtained from the calculations of HCB adsorption on cation-exchanged montmorillonite models evidenced that for the HCB adsorption, ionic radius of cations is an important factor. The calculated *E*_ads_ showed the strongest adsorption of HCB with Na^+^ and Ca^2+^ cations (− 76 and − 96 kJ mol^−1^, respectively). A significant decrease of the adsorption energy was observed for alkali metals with higher atomic mass (down to − 24 kJ mol^−1^ for Cs-modified montmorillonite), thus, having larger *IR*. The straightforward correlation between experimental log *K*_d_ values and calculated adsorption energies was not observed as the experiments were conducted in solution, whereas the calculations correspond to gas phase adsorption (interaction of isolated HCB molecule with (001) surface of montmorillonite layer) without considering the solvent effect. Additional calculations on the molecular HCB-M complexes including the solvent effect showed that solvation is a strongly destabilizing factor due to the strong hydration energy of cations. Generally, the strength of HCB adsorption to clay mineral surfaces is a result of the balance and interplay of *IR* and hydration enthalpy of exchangeable cations.

## Supplementary Information

Below is the link to the electronic supplementary material.

Supplementary file1 (PDF 228 KB)

## Data Availability

Essential data are presented in the article and supplementary material. Raw data are available on reasonable request.
