# Supplementary material for: Adsorption of the hydrophobic organic pollutant hexachlorobenzene to phyllosilicate minerals
Source: Environ Sci Pollut Res Int. 2022 Dec 23;30(13):36824–37. doi: 10.1007/s11356-022-24818-4 (PMC10039842; doi:10.1007/s11356-022-24818-4)
Supplement: Supplementary file 1 — Supplementary file1 (PDF 228 KB) [file 11356_2022_24818_MOESM1_ESM.pdf]

Supplementary Material

Adsorption of the hydrophobic organic pollutant hexachlorobenzene to phyllosilicate minerals

Leonard Böhm,[1,] Peter Grančič,[2] Eva Scholtzová,[3] Benjamin J. Heyde,[1] Rolf-Alexander Düring,[1] Jan Siemens,[1] Martin H. Gerzabek,[2] and Daniel Tunega[2]*

[1] Institute of Soil Science and Soil Conservation, Research Centre for BioSystems, Land Use and Nutrition (iFZ), Justus Liebig University Giessen, Heinrich-Buff-Ring 26, 35392 Giessen, Germany

[2] Institute for Soil Research, Department of Forest and Soil Sciences, University of Natural Resources and Life Sciences Vienna, Peter-Jordan-Straße 82, 1190 Vienna, Austria

[3] Institute of Inorganic Chemistry, Slovak Academy of Sciences, Dúbravská cesta 9, 845 36 Bratislava 45, Slovakia

* Corresponding author: leonard.boehm@umwelt.uni-giessen.de

Environmental Science and Pollution Research

25 November 2022

Summary: 4 pages including experimental and analytical details (4 tables and 1 figure)

Table S1 Physico-chemical properties of the test substance and its adsorption to organic matter

Analyte	Structure	CAS RN	Molar mass [g mol^{-1}]	Water solubility at 25 °C [µg L^{-1}] [a]	Vapor pressure at 25 °C [µm Hg] [a]	Henry's Law Constant at 25 °C [atm m^3 mol^{-1}] [a]	log K_{OW} [a]	log K_d [b] log K_{TOC} [b]
HCB	C_6Cl_6	118-74-1	284.78	6.2	0.018	0.0017	5.73	4.5–4.7 [c] 4.9–5.1

[a] SRC FatePointers Search Module, PHYSPROP database; [b] Böhm et al. (2016); [c] for organic matter with a carbon content of 29–50 %

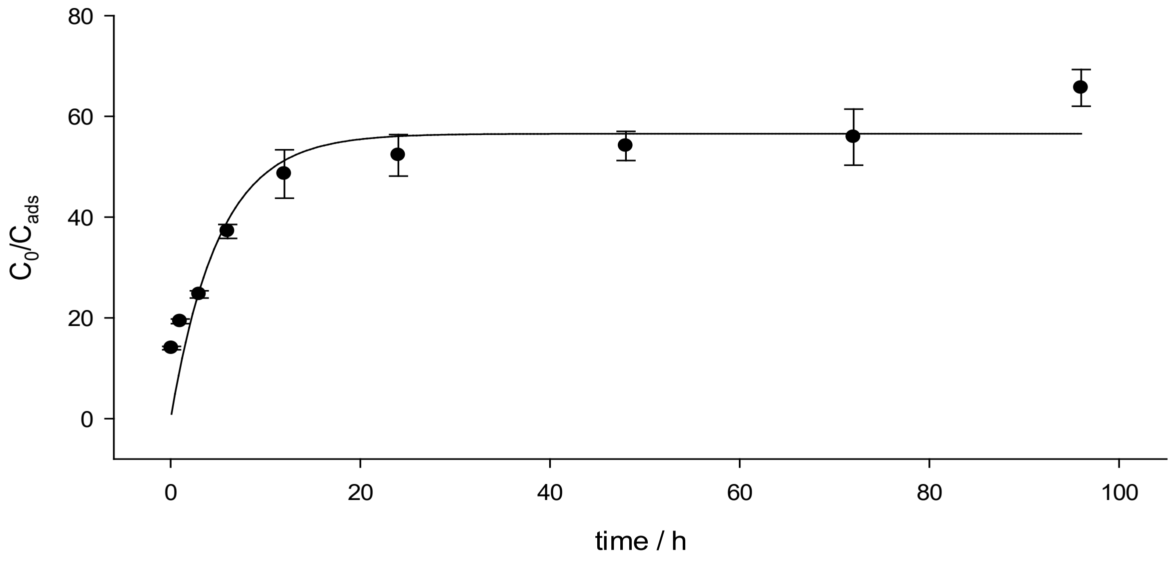

Figure S1 Estimation of the adsorption kinetic (time to equilibrium) for HCB–clay mineral systems based on an experiment with chlorite, with calculated time to equilibrium ($t_{95\%}$) = 15.2 h. Points show adsorption by quotients of C_0 (freely dissolved HCB concentration in a system without sorbent) divided by C_{ads} (freely dissolved HCB concentration in a system including sorbent)

GC-MS analysis – Text and Table S2

Measurement of HCB generally follows the methodology given in Böhm et al. (2016), Böhm et al. (2017), and Wiltschka et al. (2020). Samples were measured by gas chromatography coupled to ion trap mass spectrometry (GC-MS). The GC (Thermo Trace GC Ultra) was equipped with (a): a 30 m × 0.25 mm fused silica capillary column of the DB5 type with 0.25 µm coating (Thermo, TG-5HT) or rather (b): with a 60 m

× 0.25 mm fused silica capillary column with 0.25 µm coating (TraceGOLD TG-XLBMS, Thermo Fisher Scientific), depending on further laboratory needs. Helium 5.0 was used as carrier gas with a flow rate of 1.0 mL min^{-1}. The split/splittless injector was heated at 280 °C with a splitless time of 3 min. The transfer line to the MS was heated at (a): 290 °C or rather (b): 260 °C. The MS source was heated at 200 °C. The MS was used in selected ion storage (SIS) mode (designated as "selected ion monitoring", SIM, in Thermo Xcalibur software). Ionization of molecules was performed by electron impact ionization (70 eV). The mass used for quantification of HCB was 284 [m/z].

Table S2 Temperature program of the GC oven (retention time of HCB: (a) 11.20 min and (b) 28.13 min; total times: (a) 30.96 min and (b) 30.75 min; flow rate: 1.0 mL helium min^{-1} each)

(a)	Rate [°C min^{-1}]	Temperature [°C]	Hold time [min]
	-	60	3.00
	20	200	0.00
	3	240	0.00
	40	290	5.00
	40	305	1.00

(b)	Rate [°C min^{-1}]	Temperature [°C]	Hold time [min]
	-	40	3.00
	8	150	0.00
	4	180	0.00
	40	280	4.00

[a] TG-5HT, 30 m x 0.25 mm x 0.25 µm (Thermo); [b] TG-XLBMS, 60 m x 0.25 mm x 0.25 µm (Thermo)

Table S3 Specific surface area (SSA) of native minerals analyzed by BET (N_2) method

Mineral	Origin [a]	SSA [m^2 g^{-1}]
Kaolinite (low-defect)	CMS, KGa-1b	12
Kaolinite (high-defect)	CMS, KGa-2	21
Smectite (Ca-montmorillonite)	CMS, STx-1b	96
Smectite (montmorillonite "Cheto")	CMS, SAz-2	83
Smectite (Na-rich montmorillonite)	CMS, SWy-3	35
Smectite („calcigel")	Bavaria, Germany	n.d.
Smectite (Al-enriched nontronite)	CMS, NAu-1	77
Smectite (Al-poor nontronite)	CMS, NAu-2	47
Hectorite	CMS, SHCa-1	56
Illite	CMS, IMt-2	22
Vermiculite	Transvaal (ZA)	46
Chlorite (ripidolite)	CMS, CCa-2	14

[a] US Clay Mineral Society

Table S4 Specific surface area (SSA) of the cation modified mineral STx-1b analyzed by BET (N_2) method

$M^{+/2+}$	SSA [$m^2\ g^{-1}$]
Li-Mnt	80
Na-Mnt	78
K-Mnt	70
Rb-Mnt	58
Cs-Mnt	61
Mg-Mnt	73
Ca-Mnt	58
Sr-Mnt	54
Ba-Mnt	61

References

Böhm L, Schlechtriem C, Düring R-A (2016) Sorption of highly hydrophobic organic chemicals to organic matter relevant for fish bioconcentration studies. Environ. Sci. Technol. 50(15):8316–8323. doi: 10.1021/acs.est.6b01778

Böhm L, Düring R-A, Bruckert H-J, Schlechtriem C (2017) Can solid-phase microextraction replace solvent extraction for water analysis in fish bioconcentration studies with highly hydrophobic organic chemicals? Environ. Toxicol. Chem. 36(11):2887–2894. doi: 10.1002/etc.3854

SRC, *FatePointers Search Module: PHYSPROP database*. Data was accessed from the meanwhile inactive website *http://esc.syrres.com/fatepointer/search.asp*. The *PHYSPROP* database is still available as part of the US EPI Suite: US EPA. Estimation Programs Interface Suite™ for Microsoft® Windows, v 4.11. United States Environmental Protection Agency, 2017, Washington, DC, USA.

Wiltschka K, Neumann L, Werheid M, Bunge M, Düring R-A, Mackenzie K, Böhm L (2020) Hydrodechlorination of hexachlorobenzene in a miniaturized nano-Pd(0) reaction system combined with the simultaneous extraction of all dechlorination products. Appl. Catal. B: Environ. 275:119100. doi: 10.1016/j.apcatb.2020.119100
